# Combination of anti-PD-1 therapy and stereotactic radiosurgery for a gastric cancer patient with brain metastasis: a case report

**DOI:** 10.1186/s12885-017-3906-0

**Published:** 2018-02-12

**Authors:** Min-joo Ahn, Kanghan Lee, Kyung Hwa Lee, Jin Woong Kim, In-Young Kim, Woo Kyun Bae

**Affiliations:** 1 Departments of Internal Medicine, Chonnam National University Medical School, Gwangju, 501-757 South Korea; 2 Departments of Pathology, Chonnam National University Medical School, Gwangju, 501-757 South Korea; 3 Departments of Radiology, Chonnam National University Medical School, Gwangju, 501-757 South Korea; 4 Departments of Neurosurgery, Chonnam National University Medical School, Gwangju, 501-757 South Korea; 5 Chonnam National University Hwasun Hospital, 160 Ilsim-ri, Hwasun-eup, Hwasun-gun 519-809 South Korea

**Keywords:** Anti-PD-1 therapy, Stereotactic radiosurgery, Gastric cancer, Brain metastasis

## Abstract

**Background:**

Brain metastases from gastric cancer are difficult to treat and their prognosis is poor. Despite various possible treatments, the survival rate of such patients is still unsatisfactory; therefore, new treatment modalities or combinations of therapies need to be explored.

**Case presentation:**

We herein discuss a case of a 38-year-old man initially diagnosed with a gastric cancer brain metastasis. At first, only stereotactic radiosurgery (SRS) was performed, but it was not effective. After the brain and systemic metastases progressed, SRS and anti-PD-1 therapy were administered in combination, and the brain and intra-abdominal metastatic lesions responded satisfactorily.

**Conclusion:**

The combination of anti-PD-1 therapy and SRS could be effective against gastric cancer with brain metastases.

## Background

Brain metastases from gastric cancer are uncommon, being diagnosed in fewer than 1% of gastric cancer patients; therefore, the standard treatment has not yet been established [1]. Almost all brain metastases from gastric cancer are observed in advanced-stage disease with concurrent metastasis to other organs. In most cases, the aim of treating metastases is to achieve an appropriate relief of symptoms and assure a good quality of life [2, 3].

PD-1 is a negative co-stimulatory receptor expressed mainly on activated T cells, and downregulates excessive immune responses by binding to its ligands, PD-L1 and PD-L2. PD-L1 is constitutively expressed in various tissues and several kinds of malignancies, including gastric cancer. Binding of PD-1 to PD-L1 inhibits effector T-cell function, thus resulting in suppression of antitumor response and neoplastic growth. Several studies suggested that PD-L1 expression is significantly upregulated following *Helicobacter pylori* infection and that the resulting decrease in T-cell proliferation can be reversed with anti-PD-L1 antibodies. PD-L1 overexpression was observed in more than 40% of human gastric cancer samples and has been associated with a poor prognosis in several studies [4].

Surgery or stereotactic radiosurgery (SRS) and additional whole brain radiation therapy (WBRT) can be used for patients with a good performance status. However, the role of chemotherapy and radiosensitizers in the treatment of brain metastases from gastric cancer remains undefined. Furthermore, little is known about the safety and outcomes of patients who have received a combination of immune checkpoint blockade therapies and SRS to treat brain metastases of gastric cancer. The present report describes a case in which a patient with advanced gastric cancer with a brain metastasis was successfully treated with a combination of SRS and immune checkpoint blockade therapy.

## Case presentation

**Fig. 1:**
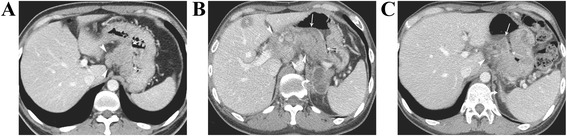
**a**. A contrast-enhanced abdominal computed tomography (CT) image shows enhancing gastric cancer (arrows) of the gastric body and conglomerate metastatic lymphadenopathies (arrowheads) along the left gastric chain. **b**. A follow-up contrast-enhanced abdominal CT image demonstrates aggravated gastric cancer of the gastric body (arrows), multiple metastatic lymphadenopathies (arrowheads) in the gastrohepatic space, a hepatic metastasis (asterisk), and left adrenal metastases (curved arrow). **c**. A follow-up contrast-enhanced abdominal CT image after anti-PD-1 therapy shows improving gastric cancer (arrows) of a swollen gastric body and markedly decreased metastatic lymphadenopathies (arrowhead). Note the resolution of the previous metastases in the left adrenal gland (curved arrow)

**Fig. 2:**
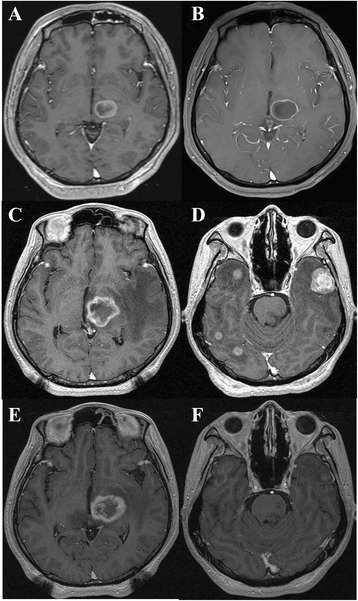
**a**. A gadolinium(Gd)-enhanced brain magnetic resonance (MR) image depicts a single metastasis (arrow) in the left thalamus. **b** and **c**. Follow-up Gd-enhanced brain MR images demonstrate tumor progression (arrow) in the left thalamus. **d**. A follow-up gadolinium-enhanced brain MR image shows several new metastases (arrows) with surrounding edema in both cerebral hemispheres. **e** and **f**. Follow-up Gd-enhanced brain MR images after anti-PD-1 therapy demonstrates the stable state of the repeatedly treated thalamic metastasis (arrow) and nearly complete resolution of the newly developed metastases

A 38-year-old male patient presented to our hospital with right side motor weakness that had started 8 months earlier. He had visited another hospital when the symptoms had started and had been diagnosed with advanced gastric adenocarcinoma with a single metastatic lesion in the left thalamus (Figs. 1a & 2a). He had undergone gamma knife radiosurgery (GKRS) at the other hospital. However, due to brain edema and deterioration of his overall condition, systemic chemotherapy had not been performed.

A physical examination revealed grade 4 motor weakness in the upper and lower right limbs. Laboratory findings revealed mild hypochromic microcytic anemia but were otherwise non-specific. Follow-up abdominal computed tomography (CT) showed aggravation of an advanced gastric malignancy with multiple metastatic regional lymph nodes, and new hepatic, left adrenal, and peritoneal metastases were also observed (Fig. 1b). Follow-up brain magnetic resonance imaging (MRI) showed a mild increase in the size of the metastasis in the left thalamus (Fig. 2b). He was only given palliative treatment and discharged.

**Fig. 3:**
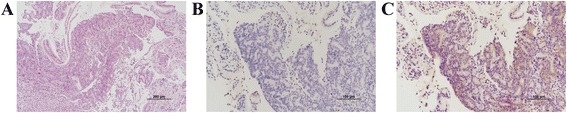
Pathologic findings of the biopsied gastric tissue. **a** The adenocarcinoma showing fused glands was intermingled with inflammatory exudates and normal mucosal glands (hematoxylin and eosin, original magnification ×100). **b** Immunohistochemistry of PD1 reveals completely negative staining in tumor cells (immunohistochemistry, original magnification ×200). **c** Tumor cells showing cytoplasmic positivity for PDL1 immunostaining (immunohistochemistry, original magnification ×200)

Five months later the patient was admitted to our neurosurgery department with a severe headache. A brain MRI showed a slight increase in the previous mass (Fig. 2c) and several newly developed metastases with surrounding edema (Fig. 2d). Repeated GKRS was performed for both recurrent and new lesions. However, his symptoms persisted and his general condition worsened. A pathological examination of the endoscopically biopsied tissue revealed moderately differentiated adenocarcinoma with glandular fusion in a cribriform pattern (Fig. 3a). By immunohistochemistry, the tumor cells were completely negative for PD1, but showed weak to moderate cytoplasmic positivity for PDL1 (Fig. 3b and c). We gave the patient an injection of pembrolizumab (Keytruda) 200 mg.

Two weeks after the injection of pembrolizumab, he returned to our hospital, reporting that his neurological symptoms had dramatically improved and that his headaches no longer occurred. He insisted that the treatment be continued, and after three doses of pembrolizumab, the patient underwent an abdominal CT and brain MRI. The abdominal CT (Fig. 1c) revealed a partial response of the gastric cancer, liver, lymph node, and brain metastases, and the brain MRI showed that the thalamic metastasis had achieved a stable state (Fig. 2e) and that there had been a dramatic reduction of the newly developed brain metastases (Fig. 2f).

The neurological symptoms were markedly improved after 3 doses of pembrolizumab. A follow-up physical examination after treatment revealed grade 3 motor weakness in his right lower limb and grade 4 motor weakness in his upper limb. Although a new brain lesion developed after 7 months of pembrolizumab treatment, his neurological symptoms and signs were not aggravated and he is being treated with systemic chemotherapy and pembrolizumab. The patient is currently still alive and in fair general condition 26 months after the initial diagnosis.

## Discussion

Therapy for a metastatic brain tumor includes surgical resection, chemotherapy, and radiation. SRS has become the main therapy for metastatic brain tumors [5]. However, the response to treatment is poor in gastric cancer patients with brain metastasis. Most brain metastases from gastric cancer are detected in the advanced stages of the disease [1, 6]. Therefore, only conservative treatment is performed in many patients. However, appropriate treatment is needed to improve the neurological symptoms of brain metastasis if the progression in other organs can be controlled.

Preclinical data suggest that PD-L1 expression is significantly upregulated following *Helicobacter pylori* infection and PD-L1 expression has been detected in more than 40% of human gastric cancer samples [4]. Furthermore, anti-PD-1 antibody pembrolizumab treatment showed a 22% overall response in patients with PD-L1-positive recurrent or metastatic adenocarcinoma of the stomach [7]. In our case, since the patient’s performance status was poor, he did not receive systemic chemotherapy. Therefore, we checked for anti-PD-L1 expression and selected a combination of SRS and anti-PD-1 therapy. As a result, his neurological function and PS recovered dramatically.

Based on our experience, we believe that a combination of SRS and anti-PD-1 therapy is useful for a brain tumor from gastric cancer. Immune checkpoint inhibitors are revolutionizing the ability to treat metastatic cancer [8]. The interaction between immune checkpoint inhibitors and standard treatments for brain metastases, such as SRS, remains under-investigated [9]. Several retrospective single-institution studies have suggested that ipilimumab in combination with radiation therapy may be more effective than radiotherapy alone for melanoma brain metastases. Knisely et al. reviewed the outcomes of 77 patients with melanoma brain metastases who received SRS as well as ipilimumab. Patients who received combination therapy demonstrated improved survival compared with those who received SRS alone [10]. Another retrospective analysis demonstrated an overall survival benefit of 19.9 months with combination therapy versus 4.0 months for SRS alone, with no associated increase in toxicity after the addition of ipilimumab to SRS [11].

At this point, however, little is known about the safety and outcomes in patients who have received immune checkpoint inhibitors and SRS to treat brain metastases of gastric cancer. In this case, the metastatic lesions of the brain initially showed no response to SRS but showed a partial response after SRS was combined with anti-PD-1 therapy. This effect may be construed as the effect of anti-PD-1 therapy alone, regardless of its combination with SRS. However, several studies have concluded that radiation therapy and immune checkpoint blockade treatment have a synergistic effect on the brain metastases of melanoma and non-small cell lung cancer. The possibility that irradiation can induce neoantigen presentation and upregulate PD-L1 expression has been previously asserted [12].

Here, we present a case in which a patient with advanced gastric cancer and brain metastasis was treated with SRS and anti-PD-1 therapy. The metastatic lesions of the brain showed a partial response after three cycles of anti-PD-1 therapy and SRS. The patient maintained excellent performance status and showed no signs of neurologic toxicities. To the best of our knowledge, this is the first case that reports the successful treatment of a brain metastasis of gastric origin through anti-PD-1 therapy combined with SRS. This case report suggests that the combination of SRS and anti-PD-1 therapy may be an option for patients with gastric cancer who have brain metastases. The main limitation of this study is its retrospective nature. Larger prospective studies are needed to determine if this combination treatment is effective for patients with SRS-unresponsive brain metastases in gastric cancer.

## Conclusion

The combination of anti-PD-1 therapy and SRS could be effective against gastric cancer with brain metastases.

## Abbreviations

CT: Computed tomography
GKRS: Gamma knife radiosurgery
MRI: Magnetic resonance imaging
SRS: Stereotactic radiosurgery
WBRT: Whole brain radiation therapy
